# N-Propanol Dehydration with Distillation and Pervaporation: Experiments and Modelling

**DOI:** 10.3390/membranes12080750

**Published:** 2022-07-30

**Authors:** Andras Jozsef Toth

**Affiliations:** Department of Chemical and Environmental Process Engineering, Environmental and Process Engineering Research Group, Budapest University of Technology and Economics, Műegyetem rkp. 3, H-1111 Budapest, Hungary; andrasjozseftoth@edu.bme.hu; Tel.: +36-1-463-1490

**Keywords:** hydrophilic pervaporation, n-propanol dehydration, mathematical modelling, parameter estimation, professional flowsheeting environment

## Abstract

This work is motivated by a fine chemical industry task where n-propanol should be separated from its aqueous mixture. To accomplish this problem, the pervaporation process intends to apply PERVAP™ 1201 type dehydration membranes and to obtain information about the water removal from an aqueous mixture of n-propanol. Different evaluation parameters (selectivities, separation factors, and total fluxes) were experimentally determined. First in the literature, this binary system’s Membrane Flash Index (MFLI) is also determined, confirming the efficiency of pervaporation against flash distillation. The experimental data from pervaporation measurements were evaluated with the improved model by Szilagyi and Toth. It has been established that the model can also be used for this case. The hybrid distillation and pervaporation system is rigorously modelled in a professional flowsheet environment (ChemCAD) and optimized with the dynamic programming optimization method. The distillation-based hybrid method without an extra added extractive agent for separating the n-propanol–water mixture has not yet been published in this computer program. The main objective functions of the hybrid method are the number of minimal theoretical stages and the minimal membrane area. It can be concluded that the process can dehydrate n-propanol with a purity of 99.9 percent.

## 1. Introduction

Separating non-ideal mixtures is a challenging task in the fine chemical industry. Pervaporation (PV) can be classified as a newer membrane method with possible industrial applications for separating non-ideal liquid mixtures forming an azeotrope [1,2]. The procedure is mainly used for dehydration of organic mixtures, removal of organics from aqueous mixtures and separation of organic–organic mixtures [3,4]. Pervaporation has specialities such as a high separation effect, simpe actualization, no pollution, and energy saving qualities, which are difficult to obtain in many cases by traditional separation processes, e.g., distillation. During the separation process, the mixture that has to be treated is vaporised on the downstream side of the PV membranes at a low pressure; in many cases under vacuum pressure [5,6].

Depending on the permeating component, pervaporation can be divided into two areas: hydrophilic PV and organophilic PV. For the dehydration tasks, hydrophilic membranes are applied. Several different quantities and factors can describe the efficiency of the PV separation process. The flux is calculated by Equation (1) [6]:

(1) $$J_{i}=\frac{P_{i}}{\Delta t\;\cdot \;A}$$

where A is the membrane area, Δt is the time of duration of the separation process, and $P_{i}$ is the amount of component i in the permeate product. The separation factor is determined by applying the following equation [6]:

(2) $$\alpha =\frac{y_{i}\left(1-x_{i}\right)}{x_{i}\left(1-y_{i}\right)}$$

where α is the separation factor (dimensionless), $x_{i}$ is the weight fraction (or concentration) of water in the feed product, and $y_{i}$ is the weight fraction (or concentration) of water in the permeate product. The pervaporation separation index (PSI) is calculated by Equation (3) [6]:

(3) $$PSI=J\cdot \left(\alpha -1\right)$$

The permeance can describe the achievement of PV membranes as a component flux normalized for driving the pressure difference-normalized flux [6,7,8]:

(4) $$\frac{P_{i}}{\delta }=\frac{J_{i}}{\gamma_{i1}\;\cdot \;x_{i1}\cdot p_{i0}-y_{i}\;\cdot \;p_{3}}$$

The selectivity of pervaporation separation is defined as the ratio of permeances [6,7,8]:

(5) $$\beta =\frac{P_{i}/\delta }{P_{j}/\delta }$$

The Membrane Flash Index (MFLI) evaluates the separation efficiency of pervaporation and flash distillation processes [9,10]:

(6) $$MFLI=\frac{y_{i}^{PV}}{y_{i}^{D}\left[VLE\right]}$$

where $y_{i}^{PV}$ is the concentration in the permeate product and $y_{i}^{D}\left[VLE\right]$ is the equilibrium distillation concentration.

The n-propanol (NPA) dehydration is the actual assignment of this work. Mostly, isopropanol–water separations are found in the literature, whereas there are far fewer references for separating the n-propanol–water mixture. Table 1 compares measured (experimental) data for dehydration of the n-propanol–water mixture. It can be seen that mainly polymeric membranes [11,12,13] have been used to separate such a mixture.

It can be determined that several membranes are suitable for separation, as seen from the high separation factor and PSI values. However, it is essential to emphasize that the complete separation of n-propanol–water is a difficult task due to the limited solubility, and it cannot be separated by conventional distillation. The n-propanol forms an azeotrope with water; the azeotropic point is c.a. 70.8% NPA–29.2 water.

Wu et al. [20] developed a hybrid extractive distillation and pervaporation method for the dehydration of n-propanol. The suggested process proved to be efficient and economical. Up to 99.9% of product purities can be achieved by adding glycerol as the extractive agent. Wang et al. [21] introduced energy-saving distillation and pervaporation methods for separating n-propanol, acetonitrile, and water–ternary mixture. The recommended method is also an extractive distillation with glycerol entrainer.

A common disadvantage of the methods shown is that an additional material to be separated is added to the system with the added extractive agent, which must also be regenerated later. Thus, the goal of this research was to develop a separation process that involves only conventional distillation and pervaporation.

## 2. Materials and Methods

Pervaporation can be considered a competing treatment alternative to distillation [6,22]. The target of this work is to examine the separation of an n-propanol–water mixture with a hybrid distillation and hydrophilic pervaporation method in a professional flowsheeting environment.

**Figure 1:**
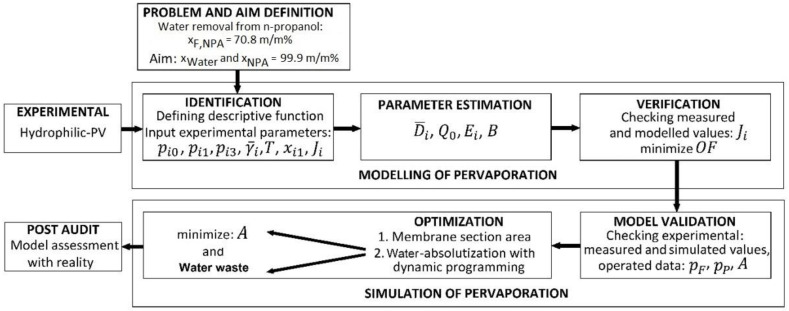
Flowchart of modelling and simulation of hydrophilic pervaporation in the case of n-propanol dehydration (based on the flowchart of Haaz and Toth [6]).

Figure 1 introduces the algorithm for the modelling and simulation of n-propanol dehydration with hydrophilic PV. This flowchart also presents the background of the semi-empirical PV model improvement, which was demonstrated in more detail in the paper by Toth et al. [6]. Firstly, the problem and the aim must be specified. In the case of hybrid systems, if possible, the size of the membrane required for separation must be reduced for economic reasons. Therefore, the purest possible product must be prepared by distillation [23]. Consequently, a 70.8 m/m% n-propanol–29.2 m/m% water mixture (azeotropic point) should be separated with hydrophilic pervaporation, and 99.9 m/m% product purity should be achieved for the water.

The modelling of the pervaporation part consists of 3 main steps, as follows: identification, parameter estimation, and verification. The pervaporation model of Szilagyi and Toth [1] was elected, which is a semi-empirical PV model. In this case, parameter estimation from experimental data is necessary to determine the parameters of the pervaporation model. The next step was the verification of determined parameters in a way that measured and modelled data were compared. Finally, rigorous modelling in a flowsheeting environment can be feasible, if the model parameters are accurate and appropriate. The ChemCAD program was used for modelling hydrophilic PV.

At first, model validation is necessary when entering the second central simulation part. The flowsheet software must examine with experimental (measured) conditions. If the results are proper, then the optimization process can be fulfilled. In the case of the n-propanol–water separation, the effective membrane area (A) has to be found [6].

### 2.1. Hydrophilic Pervaporation Experiments

**Figure 2:**
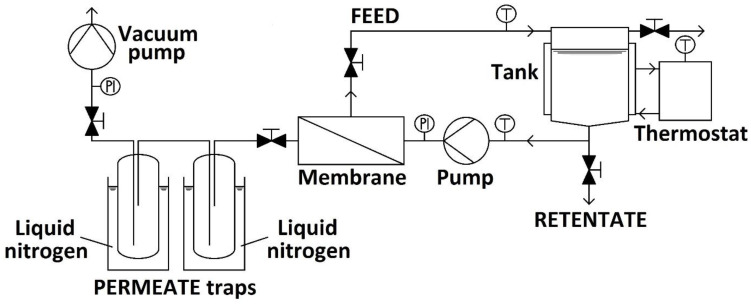
CM-Celfa P-28 Membrantechnik AG apparatus in the pervaporation setting [7,24].

The PERVAP™ 1201 Polyvinyl alcohol (PVA) composite flat-sheet PV membrane was used in the hydrophilic experiments. This type of membrane can tolerate a maximum of 50 m/m% water in the feed stream and operate at a maximum temperature of 100 °C. The experimental device was P-28 membrane apparatus from CM-Celfa Membrantechnik AG (see Figure 2) with 28 cm^2^ effective membrane area (A). The capacity of the feed tank was 0.5 L (500 mL). Cross-flow type circulation was realized at the permanent value of ∼182 L/h [7].

The vacuum was maintained at 3 mbar (2 Torr) on the side of the permeate product with a VACUUMBRAND PC2003 VARIO vacuum pump. An ultrathermostat adjusted the isothermal conditions. The permeate product was collected in two connected traps and cooled with liquid nitrogen to avoid loss of the permeate [7].

The n-propanol content of the feed (F), permeate (P), and retentate (R) were examined with Shimadzu GC2010Plus+AOC-20 gas chromatograph with CP-SIL-5CB column connected to a flame ionization detector. The water concentration was examined with Hanna HI 904 coulometric Karl Fischer titrator [7,25].

The PV experiments were carried out at three temperatures and five different concentrations, as follows: 70, 80, and 90 °C, and 32, 35, 38, 41, and 43 m/m% water content in the feed. It must be mentioned that this is the concentration range close to the azeotropic point of the n-propanol–water mixture.

### 2.2. Modelling of Pervaporation

Several models and phenomena in the literature can be used to describe the transport processes of pervaporation, such as the pore-flow model, total solvent volume fraction model, and solution–diffusion model [26,27,28,29]. One of the most widespread theories is the solution–diffusion model, which is suitable for two-layered composite PV membranes [1]. The widely used Rautenbach model [30] is also based on this theory:

(7) $$J_{i}=\frac{1}{1+\left[\frac{{\bar{D}}_{i}}{Q_{0}\;\cdot \;p_{i0}\;\cdot \;{\bar{\gamma }}_{i}}\right]}\cdot \frac{\bar{D_{i}}}{\bar{\gamma_{i}}}\cdot \left(\frac{p_{i1}-p_{i3}}{p_{i0}}\right)\;\;\;\;\;\;\;i=\left(1,\ldots ,k\right)$$

The procedure by Szilagyi and Toth [1] was elected for modelling hydrophilic PV in the present work. The essential, original equation of this model:

(8) $$J_{i}=\frac{1}{1+\left\{\frac{\left[{\bar{D}}_{i}\;\cdot \;exp\left(x_{i1}{}^{B}\right)\right]}{\left(Q_{0}\;\cdot \;p_{i0}\;\cdot \;{\bar{\gamma }}_{i}\right)}\right\}}\cdot \frac{\left[{\bar{D}}_{i}\;\cdot \;exp\left(x_{i1}{}^{B}\right)\right]}{{\bar{\gamma }}_{i}}\cdot \left(\frac{p_{i1}-p_{i3}}{p_{i0}}\right)\;\;\;\;\;\;\;i=\left(1,\ldots ,k\right)$$

This pervaporation model is a development of the basic Rautenbach model [30] and the model of Valentinyi et al. [2]. The advancements consider the concentration dependencies of the transport coefficient ($\bar{D_{i}}$) and the temperature dependencies of the pervaporation [7,31].

It must be mentioned that the porous supporting layer’s permeability coefficient (Q_0_) was infinitely big compared to the $\bar{D_{i}}$ which made this layer’s resistance negligible. Therefore the first part of Equation (7) and Equation (8) can be ignored [31]. The models, as mentioned earlier, were simplified in the following way during practical calculations:

(9) $$J_{i}=\frac{\bar{D_{i}}}{\bar{\gamma_{i}}}\cdot \left(\frac{p_{i1}-p_{i3}}{p_{i0}}\right)$$

(10) $$J_{i}=\frac{{\bar{D}}_{i}\;\cdot \;exp\left(x_{i1}{}^{B}\right)}{{\bar{\gamma }}_{i}}\cdot \left(\frac{p_{i1}-p_{i3}}{p_{i0}}\right)$$

Equation (9) as Model 1 and Equation (10) as the improved model (Model II) were applied to model the measurement data.

Partial pressures ($p_{i0}$) were computed according to the Antoine equation [6]:

(11) $$p_{i0}=exp\left[A+\frac{B}{T}+ClnT+DT^{E}\right]*10^{-5}$$

where A, B, C, D and E are substance-dependent constants. The transport coefficient depends on the temperature in an exponential way of Arrhenius type [6]:

(12) $${\bar{D}}_{i}={\bar{D}}_{i}^{*}exp\left[\frac{E_{i}}{Ʀ}\left(\frac{1}{T^{*}}-\frac{1}{T}\right)\right]$$

$E_{i}$ is the activation energy for component i, and is associated with the transport coefficient, and $T^{*}\;$ is the reference temperature, equal to 293 K in Equation (12). The activity coefficients can be determined with the Wilson equation or with different vapour–liquid equilibrium models. The detailed professional background of the semi-empirical pervaporation models can be found in [2,4,6], and the main knowledge of Model I and Model II can be described in a paper by Szilagyi and Toth [1].

The transport coefficients, activation energies, and in the case of Model II for both compounds, the B parameters, were estimated based on the experimental data [7]. A non-linear estimation process was applied by defining a user-specified regression custom loss function in STATISTICA^®^ software. Verification of the accuracy of the model can be achieved with objective function (OF), which minimizes the deviation of the measured and the modelled values (Equation (13)).

(13) $$OF=\overset{n}{\underset{i=1}{\sum }}{\left(\frac{J_{i,measured}-J_{i,\;modelled}}{J_{i,measured}}\right)}^{2}$$

The Model II was inspected in the case of alcohol–water separations. Methanol–water hydrophilic pervaporation was investigated in the range of 1.78–3.075 m/m% feed water concentration with Sulzer PERVAP™ 1510 from PVA membranes by Szilagyi and Toth [1]. The Sulzer PERVAP™ 1510 membrane was also investigated for pervaporation dehydration in the range of 4.57–36.39 m/m% feed water concentrations by Szilagyi and Toth [1]. The accuracy of the improved model was also confirmed in two more organophilic cases using the polymeric Sulzer PERVAP™ 4060 membrane from PDMS: isobutanol–water mixture separation, in the range of 0.5–7.0 m/m% isobutyl alcohol feed concentrations and ethanol–water separation, between 0.4 and 8.4 m/m% ethanol feed concentrations by Szilagyi and Toth [1].

### 2.3. Simulation of Hybrid Distillation and Pervaporation Method

**Figure 3:**
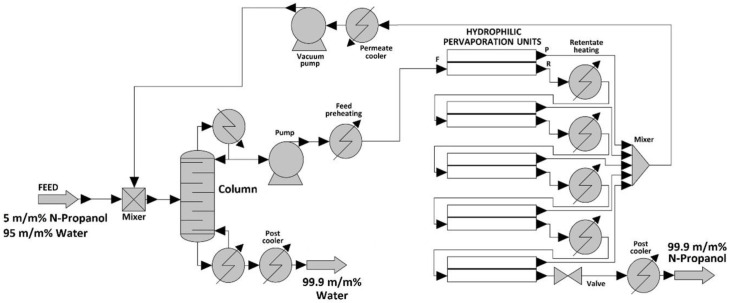
Simulated hybrid distillation and hydrophilic pervaporation method for separating n-propanol–water binary mixture.

The user-added PV unit was written for Model I and Model II applications in the ChemCAD flowsheeting program [3]. After the appropriate PV model validation (see Figure 1), the hybrid distillation and hydrophilic pervaporation system can be rigorously modelled and optimized with a dynamic programming optimization method [7,32]. The flowsheet of the hybrid distillation and hydrophilic pervaporation system can be seen in Figure 3. 1000 kg/h n-propanol–water feed flow with 5 m/m% n-propanol–95 m/m% water mixture had to be separated.

Other tools were also needed for the hydrophilic pervaporation method. Preheating the feed stream and growing its pressure was necessary, as it had atmospheric circumstances (20 °C, 1 bar). The reheating of the retentate stream had to be applied after each pervaporation system, except for the last, because adiabatic PV was used [7,23,33]. In the system, the pumps increased the pressure, and heat exchangers adjusted the system’s temperature. Permeate streams were collected, mixed, and condensed with a permeate cooler unit, and their pressure was raised again from the vacuum with the pump unit [7]. It can be seen in Figure 3 that the permeate stream was recycled at the beginning of the system, and it was mixed with feed flow. Valve and post cooler decreased the retentate product’s pressure and temperature [6,7].

The number of theoretical stages (N), the place of the feed stream, the reflux ratio, and the heat duty were optimized in the case of the distillation method. SCDS column was applied with NRTL model.

## 3. Results and Discussion

**Figure 4:**
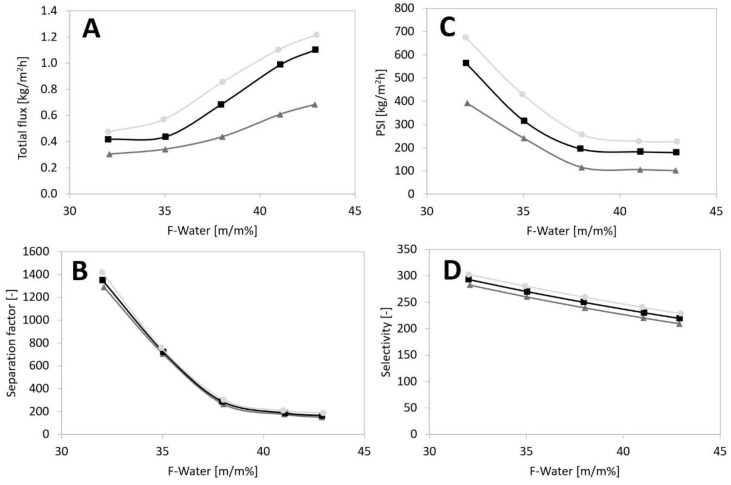
Separation achievement as a function of feed content at different operating temperatures for PERVAP™ 1201 membrane (70 °C: 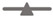; 80 °C: 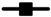; 90 °C: 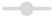), (**A**) Total flux, (**B**) Separation factor, (**C**) PSI, (**D**) Selectivity.

Figure 4 depicts the impact of feed composition on the hydrophilic pervaporation output of the PERVAP™ 1201 membranes at different feed temperatures. It can be observed that growing feed aqueous content increases the permeate (total) fluxes, and growing the temperature also increases the fluxes (see Figure 4A). However, growing the aqueous content reduces the separation factor and selectivity values (Figure 4B,D). At the aqueous feed content of 32.02 m/m% and at 90 °C the maximum separation factor value of 1420 can be reached. The PSI (Figure 4C) and the separation factor (Figure 4B) follow the tendency of the selectivity (Figure 4D). It can be noticed that PERVAP™ 1201 had the highest separation factor in the case of PVA membranes and it also had high PSI in the category of n-propanol dehydration membranes, compared to other experimental results in the literature (see Table 1).

A similar trend has already been published in the case of n-propanol separations with polymeric pervaporation membranes [15,34,35]. It can be noticed that at higher aqueous content, the separation efficacy of the pervaporation membranes progressively declined. A probable cause is that growing the aqueous concentration increases aqueous sorption through the flat-sheet pervaporation membrane [36]. As an impact, the PV membrane becomes swollen, better owing to its dehydration (hydrophilic) property. Furthermore, the swollen sheet, because of its increased free volume redounds the diffusion of n-propanol through the membrane material. As a result, the value of the separation factor decreases [6,24,37].

**Figure 5:**
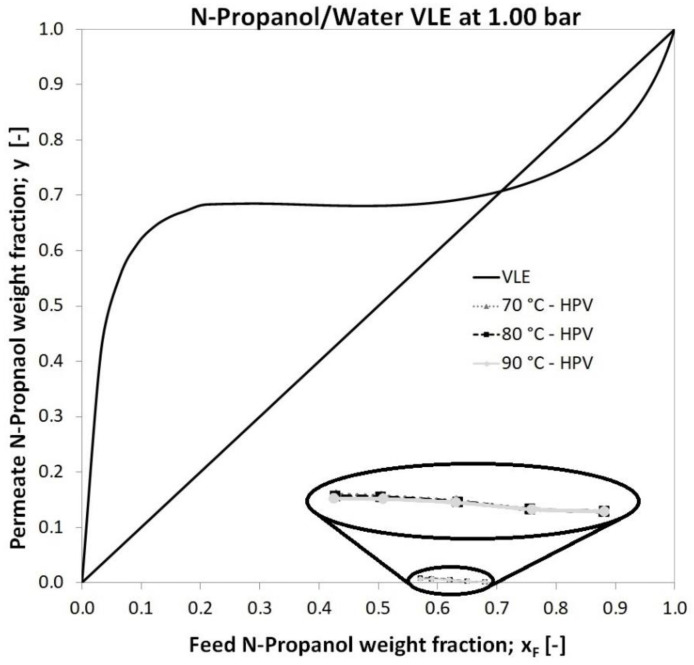
Experimental permeate n-propanol weight fractions (also enlarged version) of hydrophilic pervaporation on the n-propanol–water VLE diagram.

The feed n-propanol weight fractions for the hydrophilic pervaporation are depicted against the permeate n-propanol weight fractions in Figure 5. It can be noticed that there is no remarkable discrepancy between the permeate n-propanol weight fractions in the case of temperatures. The vapour–liquid equilibrium of the n-propanol–water binary mixture is also depicted at 1 bar (full line) so that PV and flash distillation can be compared [6,7,24]. It can be determined that the n-propanol permeate weight fractions are deep under the equilibrium vapour concentration in Figure 5, which means a very high water concentration in the permeate product. Consequently, it can be established that the dehydration pervaporation method shows remarkably better separation compared to flash distillation. Therefore PV can be a competitive option against flash distillation. This finding is also confirmed by an MFLI value higher than 1, which averaged 3.22.

Table 2 presents the values of activation energies, transport coefficients, and exponential parameters of the pervaporation models estimated by the STATISTICA^®^ program environment.

**Figure 6:**
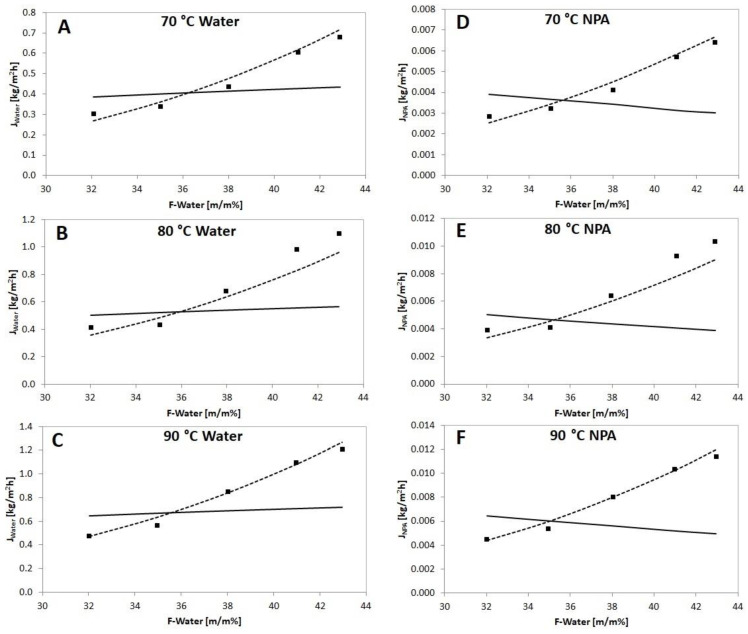
Experimental water and n-propanol fluxes (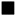) compared to calculated partial fluxes with Model I (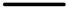) and Model II (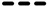) in a function of feed water concentration with PERVAP™ 1201 membrane.

Figure 6 compares the experimental and calculated partial (water and n-propanol) fluxes.

Table 3 summarizes the minimized objective functions of the two models.

It can be seen in Figure 6 and Table 3 that Model II is more accurate for the description of hydrophilic PV than the previous Rautenbach model. Model I presumes constant tendency of the transport coefficient. Consequently, the concentration dependencies of ${\bar{D}}_{i}$ need to be considered for the appropriate model application. The improved model considers the findings of many researchers, according to which an exponential relationship can be observed between feed concentration and the diffusion coefficient [6,7,24,38,39].

It has to be emphasized that in this case, only Model II is suitable for proper modelling of hydrophilic PV in a flowsheet environment. Table 4 shows the comparison of measured and calculated total fluxes obtained for the laboratory pervaporator apparatus by ChemCAD software. This can be considered a validation of the pervaporation model in the flowsheet environment. In the case of the validation simulations, the main operating conditions were as follows: the feed pressure was 3 bar, the permeate pressure was 3 mbar, and the feed temperature was 90 °C. It can be determined that the results of the experimental and model fluxes match with great accuracy.

The optimised hybrid system parameters were as follows: 40 theoretical stages with middle feed. The reflux ratio of the distillation column was 1 and the total effective membrane area was 300 m^2^. These settings achieved the desired product purities (99.9 m/m% of water and n-propanol), as seen in Figure 3.

The optimised hybrid system parameters were as follows: 40 theoretical stages with middle feed. The reflux ratio of the distillation column was 1 and the total effective membrane area was 300 m^2^. These settings achieved the desired product purities (99.9 m/m% of n-propanol and min. 99.9 m/m% of water), as seen in Figure 3. Table 5 shows the composition of the water and n-propanol product in the function of different membrane areas.

**Figure 7:**
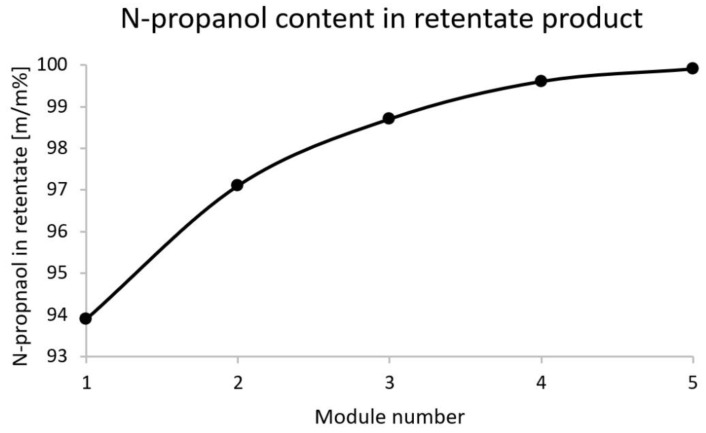
N-propanol weight percent in retentate product.

Figure 7 shows the progression of retentate n-propanol content in membrane modules (The size of 1 module is 60 m^2^).

The design alternatives’ comprehensive analysis needs to evaluate the energy demands at the different treatment units [7]. Table 6 introduces the calculated heat duties. It can be noticed that the reboiler duty has the highest heat demand, and the post cooler is almost remarkable. This observation is considered consistent with those experienced in other distillation and hydrophilic pervaporation hybrid procedures [40,41].

## 4. Conclusions

The pervaporation dehydration was investigated experimentally to explore the units describing the separation. The permeate flux of the examined PERVAP™ 1201 membrane was established to vary from 304 to 1216 g/m^2^h over the feed water content range of 32–43 m/m% at 70–90 °C feed temperature. The highest separation factor of 1420 and the second highest permeate flux (1.22 kg/m^2^h) were measured with a flat-sheet PVA membrane. It was found that PERVAP™ 1201 had a relatively high pervaporation separation index value compared to other published PSI data in the case of the n-propanol–water system. The figures represented that total flux and selectivity were in inverse relation, which was in line with the literature.

The representations of the parameter fitting of the hydrophilic PV showed that the developed model by Szilagyi and Toth [1] could more accurately estimate the experimental fluxes. The fitting of parameters for the n-propanol–water binary system was implemented for the first time in the literature, and this can be considered the main novelty of this work. Therefore, it was also suitable for the modelling of pervaporation in a flowsheet environment. A hybrid process based on conventional distillation has been developed using the improved PV model. It can be found that the investigated method was capable of dehydrating the n-propanol without adding extra components.

## Figures and Tables

**Table 1:** Summary of measured data for hydrophilic pervaporation of n-propanol–water mixture (based on, expanded [14]).

Membrane Type	T	F_water_	J_total_	α	PSI	Reference
	[°C]	[m/m%]	[kg/m^2^h]	[–]	[kg/m^2^h]	
PVA cross-linked with citric acid	30	10	0.08	141	11	Burshe et al., 1997 [15]
PVA/PAN	60	5	0.15	90	13	Gesing, 2004 [16]
αAl_2_O_3_/PVA	70	10	2.20	50	108	Peters et al., 2006 [17]
PERVAP™ 2201D (PVA/PAN)	70	10	0.52	500	259	Teleman et al., 2022 [14]
PERVAP™ 2201D (PVA/PAN)	60	10	0.26	2500	650	Teleman et al., 2022 [14]
Poly(urethane-imide)-PUI-2000	50	20	8.80	179	1566	Sokolova et al., 2018 [18]
Poly(urethane-imide)-PUI-530	50	20	5.10	437	2224	Sokolova et al., 2018 [18]
polyvinylamine/polyvinylsulphate	59	10	1.20	6000	7199	Toutianoush et al., 2002 [19]

**Table 2:** ${\bar{D}}_{i}$, $E_{i}$, and B values of n-propanol–water binary mixture estimated by STATISTICA*^®^* program environment.

PERVAP™ 1201	Model I		Model II	
	Water	NPA	Water	NPA
${\bar{D}}_{i}$ [kmol/m^2^h]	7.15 × 10^−3^	4.20 × 10^−5^	2.40 × 10^−5^	2.32 × 10^−3^
$E_{i}$ [kJ/kmol]	2.4644	2.6053	2.7707	2.9966
B [−]			8.38	−12.08

**Table 3:** Objective functions resulted in Model I and Model II.

PERVAP™ 1201	Objective Function-Water	Objective Function-NPA
Model I	1.369	2.468
Model II	0.123	0.125

**Table 4:** Comparison of experimental and model fluxes for laboratory-size separation: model validation.

F_water_	J_total_—Measured (Experiment)	J_total_—Calculated (Model)	Deviation
[m/m%]	[kg/m^2^h]	[kg/m^2^h]	[%]
32	0.48	0.48	1.4
35	0.57	0.58	1.3
38	0.86	0.85	−0.7
41	1.10	1.11	1.1
43	1.20	1.21	−0.9

**Table 5:** Composition of Water- and N-propanol products in the function of membrane area.

Membrane	Water Product (Bottom Product)		N-Propanol Product (Retentate)	
Area	Water	N-Propanol	Water	N-Propanol
[m^2^]	[m/m%]	[m/m%]	[m/m%]	[m/m%]
60	99.68	0.32	6.1	93.9
120	99.85	0.15	2.9	97.1
180	99.93	0.07	1.3	98.7
240	99.98	0.02	0.4	99.6
300	99.99	0.01	0.1	99.9

**Table 6:** Calculated heat duties of the hybrid system.

Calculated Heat Duties		Q_Heating_ [MJ/h]	Q_Cooling_ [MJ/h]
Distillation	Reboiler	501	
	Condenser		−166
	Post cooler		−317
Pervaporation	Feed preheating	1	
	Retentate heating	44	
	Permeate cooler		−53
	Post cooler	.	−9

## Data Availability

Data is contained within the article.

## Nomenclature

A	Membrane transfer area	$\left[m^{2}\right]$
B	Constant in Model II	$\left[-\right]$
D	Distillate product	
${\bar{D}}_{i}$	Transport coefficient of component i	$\left[kmol/\left(m^{2}h\right)\right]$
${\bar{D}}_{i}^{*}$	Relative transport coefficient of component i	$\left[kmol/\left(m^{2}h\right)\right]$
$E_{i}$	Activation energy of component i in Equation (12) for temperature dependence of the transport coefficient	$\left[kJ/mol\right]$
F	Feed	
i	Component number	
j	Component number	
$J_{total}$	Total flux	$\left[kg/\left(m^{2}h\right)\right]$
$J_{i}$	Partial flux	$\left[kg/\left(m^{2}h\right)\right]$
N	Number of theoretical stages	[-]
P	Permeate	
$p_{i0}$	Pure i component vapour pressure	$\left[bar\right]$
$p_{i1}$	Partial pressure of component i on the liquid phase membrane side	$\left[bar\right]$
$p_{i3}$	Partial pressure of component i on the vapour phase membrane side	$\left[bar\right]$
$p_{3}$	Pressure on the permeate side	$\left[bar\right]$
$P_{i}/\delta$	Permeance of component i	$\left[mol/\left(m^{2}\;h\;bar\right)\right]$
Q_0_	Permeability of the porous supporting layer of the membrane	[$kmol/\left(m^{2}\;h\;bar\right)$]
R	Retentate	
Ʀ	Gas constant	$\left[kJ/\left(kmol\;K\right)\right]$
T	Temperature	$\left[{}^{\circ}C\right]$
$T^{*}$	Reference temperature: 293 K	
$x_{F}$	Feed n-propanol weight fraction in y−x vapour-liquid equilibrium (VLE) diagram (Figure 5)	$\left[-\right]$
$x_{i1}$	Concentration of component i in the feed	$\left[m/m\%\right]$
y	Permeate n-propanol and water weight fraction in y−x vapour-liquid equilibrium (VLE) diagram (Figure 5)	$\left[-\right]$
$y_{i}^{PV}$	Permeate concentration in Membrane Flash Index (MFLI)	$\left[m/m\%\right]$
$y_{i}^{D}\left[VLE\right]$	Equilibrium distillation value in Membrane Flash Index (MFLI)	$\left[m/m\%\right]$

## Abbreviations

HPV	Hydrophilic pervaporation	
hydr	hydrophilic	
MFLI	Membrane Flash Index	$\left[-\right]$
NPA	n-propanol	
OF	Objective function	
PSI	Pervaporation Separation Index	$\left[kg/\left(m^{2}h\right)\right]$
PVA	Polyvinyl alcohol	
PV	Pervaporation	
VLE	Vapour-Liquid Equilibrium	
*Greek letters*		
α	Separation factor	
β	Selectivity	
${\bar{\gamma }}_{i}$	Average activity coefficient of component i	
$\gamma_{i1}$	Activity coefficient of component i in the feed	
δ	Membrane thickness	$\left[\mu m\right]$
